# Supplementary figures and images for: Genome-Wide Identification and Expression Analysis of m6A Methyltransferase Family in Przewalskia tangutica Maxim
Source: Int J Mol Sci. 2025 Apr 11;26(8):3593. doi: 10.3390/ijms26083593 (PMC12027458; doi:10.3390/ijms26083593)

Motif 1

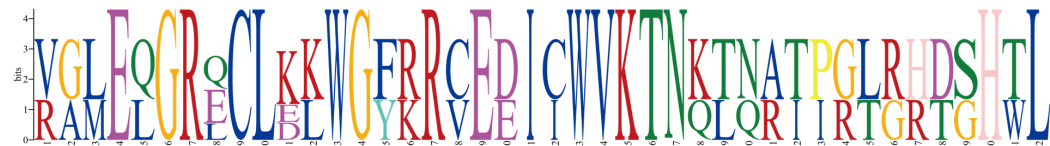

Motif 2

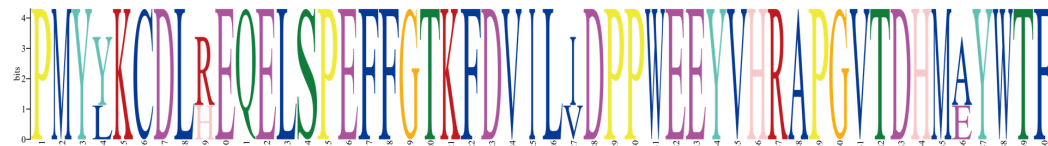

Motif 3

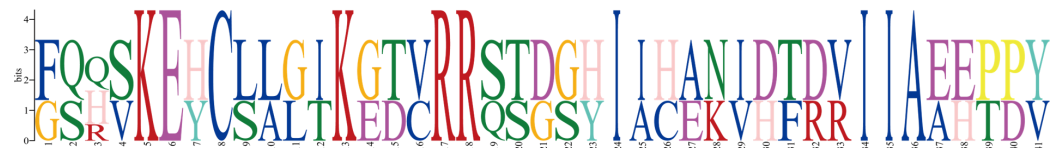

Motif 4

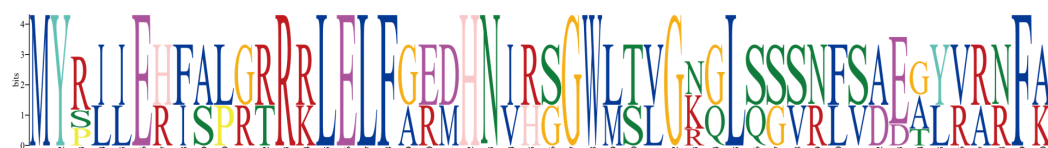

Motif 5

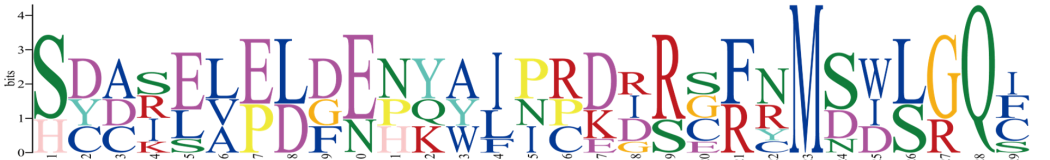

Motif 6

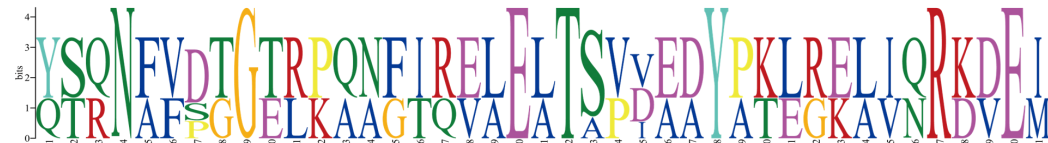

Motif 7

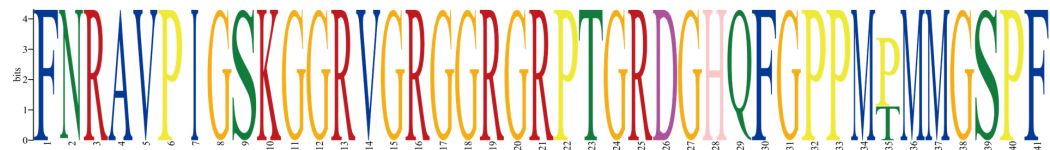

Motif 8

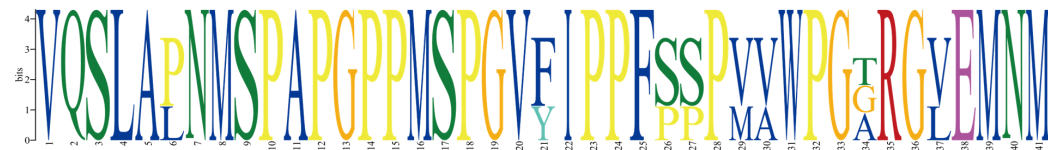

Motif 9

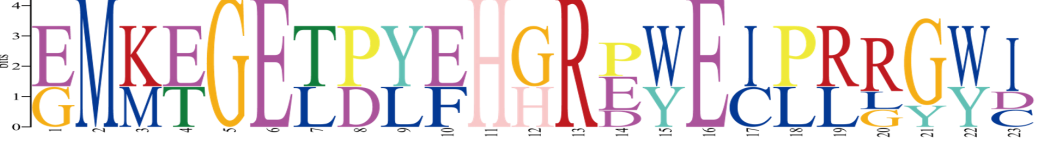

Motif 10

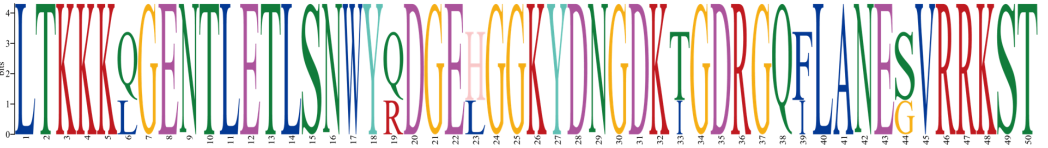

Figure S1: motif 1-10 analysis results

Supplement: Supplementary file 1 [file ijms-26-03593-s001.zip › Figure S1.pdf]

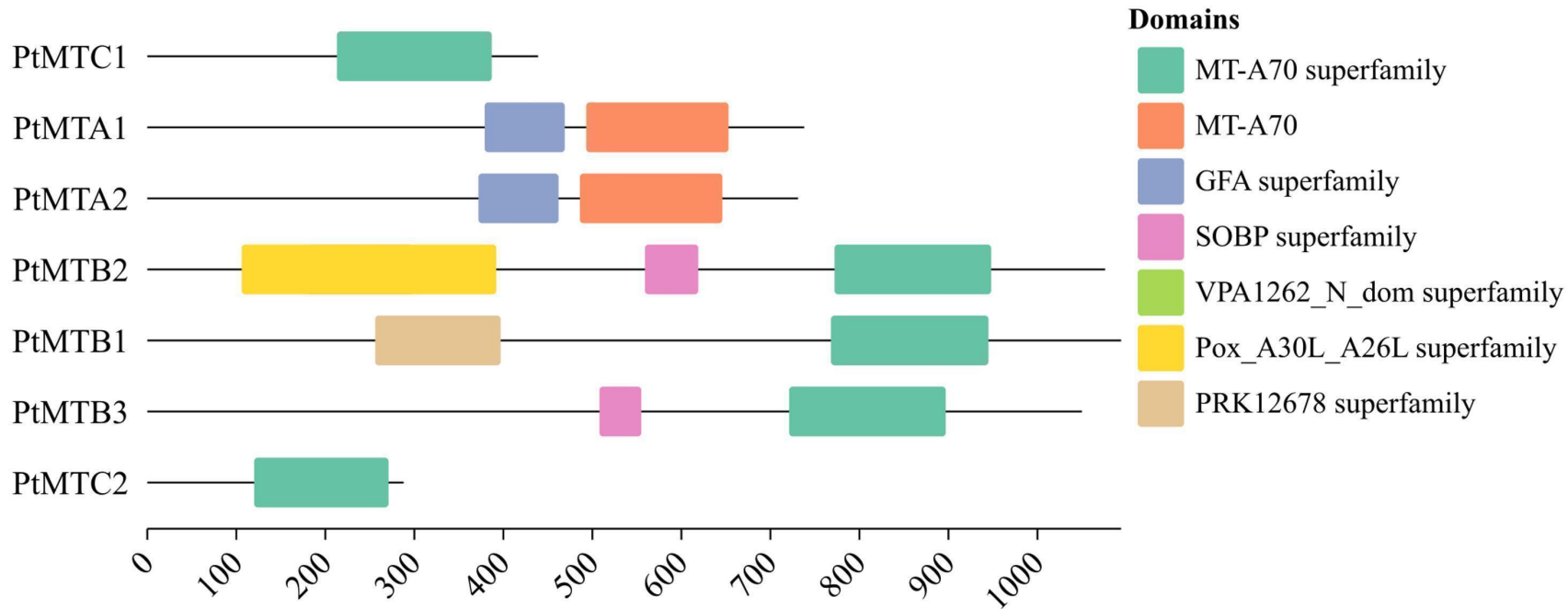

Figure S2. Conserved domain analysis

Supplement: Supplementary file 1 [file ijms-26-03593-s001.zip › Figure S2.pdf]

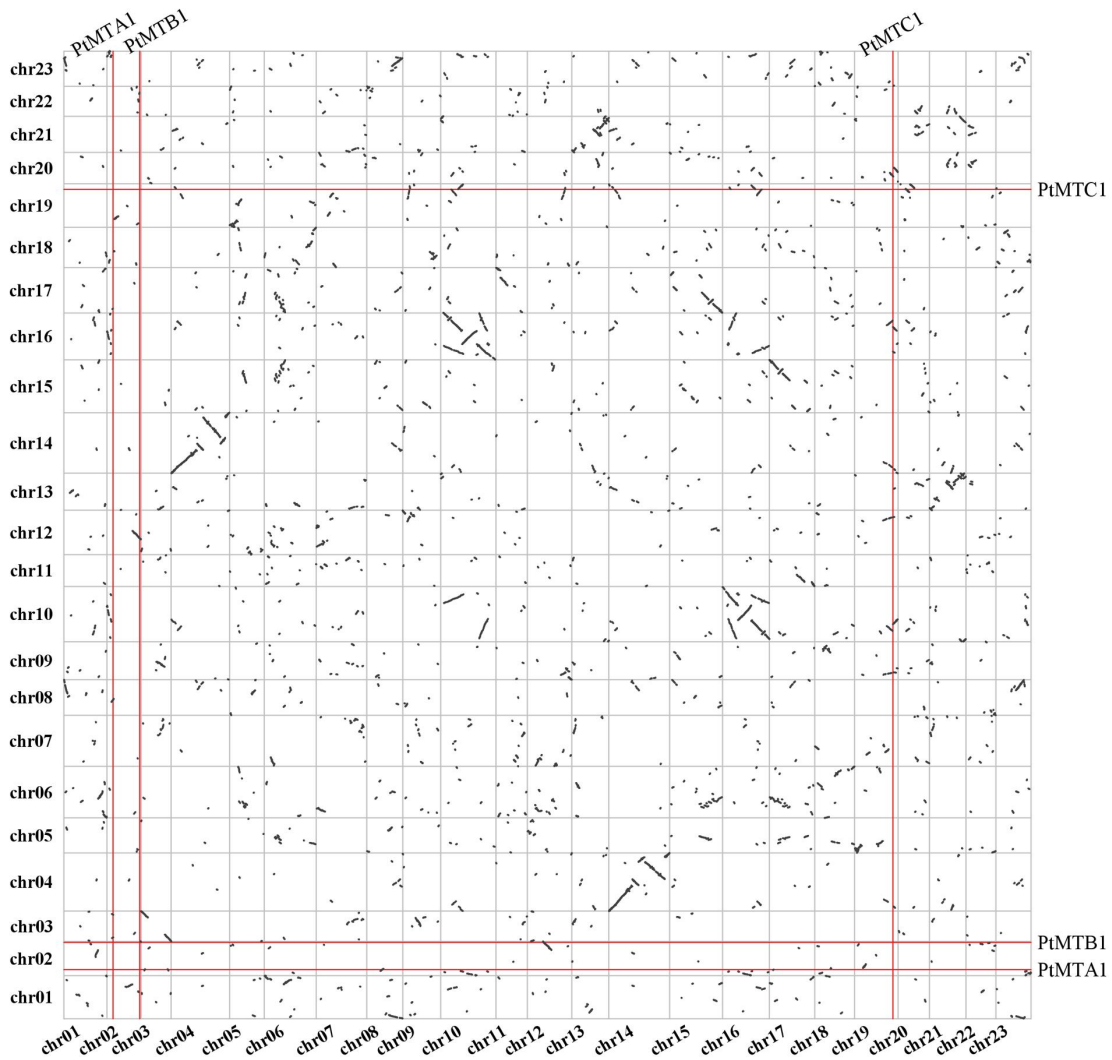

Figure S3. DotPlot

Supplement: Supplementary file 1 [file ijms-26-03593-s001.zip › Figure S3.pdf]
